# Inhalation of Medicated Vapors in Diseases of the Respiratory Organs

**Published:** 1882-09

**Authors:** 


### Abstracts.

INHALATION OF MEDICATED VAPORS IN DISEASES OF THE RESPIRATORY ORGANS.—Guillemin (*Archives Méd. Belges—Lond. Record*) summarizes his views as follows:

1. The affections of the mucous membrane of the respiratory passages may in certain cases be advantageously treated by inhalations of medicated vapors.

2. In the first stage of acute inflammation of this mucous membrane, pain, cough, and painful sensations, which are the consequence of irritation and dryness, are rapidly calmed by inhalations of warm, moist, and aromatic vapors.

3. The calming action is still more decided if to the liquid, which serves for inhalation, there be added a small quantity of certain volatile calmative substances such as ether, distilled cherry-laurel water, or conium.

4. Frequently renewed inhalations of essence of turpentine, when they are administered at the commencement of the first period of inflammation, may arrest its progress.

5. The vapor of iodine exercises an irritant action on the mucous membrane of the air-passages. It induces efforts of coughing, and augments the secretion of the mucus of the air-passages. This irritating action may be utilized: (*a.*) To diminish the swelling of the mucous membrane by causing the inflammation to pass from the first to the second stage; this indication is present especially in cases where the inflammation occupies the small bronchi; the swelling of the mucous membrane is sufficient to give rise to fear of respiratory insufficiency. (*b.*) To diminish the viscosity of the products of morbid secretion by their admixture with the mucus, of which the vapors increase the formation.

(*c*.) To induce efforts to cough, and to disembarrass the air-passages from the products which are there accumulated.

6. It is not only by its irritating properties that the vapor of iodine modifies the mucous membrane of the air-passages. Iodine in reality possesses the property of stopping purulent secretion, and, on the other hand, it arrests and prevents putrescence. Thus, when the mucous membrane of the air-passages yields a purulent secretion, resulting either from an acute inflammation in the third stage, or from a chronic inflammation, the inhalations of iodine will determine by degrees the quantity of pus, and finish in certain cases by entirely changing the nature of the secretion, which becomes completely mucous.

7. Although the essence of turpentine, in the fluid condition, is a sufficiently powerful irritant for the tissues with which it is placed in contact, inhalation of this essence is easily supported by the mucous membrane of the air-passages. It only brings on very moderate irritation, and very rarely provokes fits of coughing.

8. When the mucous membrane is affected, and yields a product of secretion, these vapors have the effect of diminishing the quantity and augmenting the consistence of this.

9. If the product of secretion be purulent, the inhalation of essence of turpentine, continued during a sufficiently long time, progressively diminishing the quantity of pus, may, in certain cases, completely stop the secretion. The inhalations are indicated in all affections of the larynx, of the trachea, and of the bronchi, when accompanied by a very copious muco-purulent secretion without viscosities. On the other hand, the use of them must be avoided whenever expectoration is difficult, in consequence of the too great viscosity of the products of secretion.

10. In cases when these products are at the same time very copious and very viscous, it is possible, by alternate inhalations of vapors of iodine and vapors of turpentine, to rapidly diminish the quantity of secretion without increasing its viscosity. The inhalation of iodine should always be used in the first instance.

11. Inhalation of essence of turpentine is indicated in hæmoptysis, and is very successful in cases of hæmoptysis of average intensity.—*Detroit Lancet.*

